# Brain relaxation using desflurane anesthesia and total intravenous anesthesia in patients undergoing craniotomy for supratentorial tumors: a randomized controlled study

**DOI:** 10.1186/s12871-023-01970-z

**Published:** 2023-01-10

**Authors:** Ze Jiang, Youxuan Wu, Fa Liang, Minyu Jian, Haiyang Liu, Hongxun Mei, Ruquan Han

**Affiliations:** Department of Anesthesiology, Beijing Tiantan Hospital, Capital Medical University, No. 119, Southwest 4Th Ring Road, Fengtai District Beijing, People’s Republic of China

**Keywords:** Desflurane, Propofol, Brain relaxation, Supratentorial tumors

## Abstract

**Background:**

Satisfactory brain relaxation is essential in neurosurgery. Desflurane anesthesia and propofol-based total intravenous anesthesia (TIVA) have different effects on cerebral hemodynamics, potentially contributing to discrepant brain relaxation. The purpose of this study was to compare the effects of desflurane and TIVA on brain relaxation in patients undergoing craniotomy for supratentorial tumors.

**Methods:**

In this randomized, controlled study, we enrolled patients aged 18–60 years, with ASA I–III, who were scheduled to undergo elective craniotomy for supratentorial tumors. Patients were randomly assigned in a 1:1 ratio to receive desflurane anesthesia or TIVA. The primary outcome was the proportion of satisfactory brain relaxation. Secondary outcomes included emergence and extubation times, recovery of cognitive function and postoperative complications.

**Results:**

Of 369 patients who were assessed for eligibility, 111 were randomized and 110 were included in the modified intention-to-treat analysis (55 in the desflurane group and 55 in the TIVA group). The proportion of satisfactory brain relaxation was similar between the two groups: 69% in the desflurane group and 73% in the TIVA group (RR: 0.950, 95% CI: 0.748–1.207; *P* = 0.675). Patients assigned to the desflurane group had shorter emergence (10 [8–13] min vs. 13 [10–20] min, *P* < 0.001) and extubation times (13 [10–18] min vs. 17 [13–23] min, *P* < 0.001), and better recovery of cognitive function at 15 min after extubation (16 [0–24] vs. 0 [0–20], *P* = 0.003), but experienced increased postoperative nausea and vomiting (PONV) (16 [29%] vs. 6 [11%] *P* = 0.017) and tachycardia (22 [40%] vs. 9 [16%], *P* = 0.006) during recovery.

**Conclusions:**

Desflurane anesthesia and TIVA provide similar brain relaxation in patients without intracranial hypertension undergoing elective craniotomy. Desflurane accelerates the recovery from anesthesia but is associated with increased PONV and tachycardia during the recovery period.

**Trial registration:**

Clinicaltrial.gov (NCT04691128). Date of registration: December 31, 2020.

**Supplementary Information:**

The online version contains supplementary material available at 10.1186/s12871-023-01970-z.

## Introduction

Satisfactory brain relaxation is essential in neurosurgery for sufficient surgical exposure and minimizing the damage to normal brain tissue [1]. Intravenous and inhalational anesthetic agents have different effects on cerebral hemodynamics, potentially contributing to discrepant brain relaxation.

Propofol-based total intravenous anesthesia (TIVA) has been widely accepted in neurosurgery due to the capacity of decreasing intracranial pressure (ICP) by reducing cerebral blood flow (CBF) and cerebral blood volume (CBV) [2]. On the contrary, inhalational anesthetics dose-dependently increase CBF by promoting cerebral vasodilatation, which may increase ICP and potentially lead to unsatisfactory brain relaxation [3]. However, several clinical studies implied that inhalational anesthesia and TIVA were associated with comparable brain relaxation in patients undergoing elective craniotomy for brain tumors [4–6], but most of them only focused on sevoflurane and isoflurane and ignored desflurane. Desflurane is an inhalational anesthetic with low blood solubility that provides rapid emergence, which may facilitate early detection of surgery-related complications, such as hematoma formation, acute cerebral infarction and neurological deficits [7]. Despite these favorable qualities, the use of desflurane in neurosurgery has been debated because of its more pronounced effect on cerebral vasodilatation [8, 9], potentially leading to unsatisfactory brain relaxation by increasing CBV.

To our knowledge, there are few clinical trials designed to evaluate desflurane anesthesia and TIVA on brain relaxation during craniotomy. Most did not take brain relaxation as a primary endpoint and failed to fully address various factors that may influence brain relaxation, such as the use of mannitol [10, 11]. Also, the sample size may be underpowered to discover the differences in brain relaxation between the two anesthesia regimens [12].

Therefore, we conducted this randomized controlled trial to test the difference between desflurane anesthesia and TIVA in providing brain relaxation in patients undergoing elective craniotomy without severe intracranial hypertension.

## Methods

### Trial design

This was a single-center, randomized, controlled, patient and outcome assessor-blinded trial. Patients were consecutively recruited from Beijing Tiantan Hospital, Capital Medical University from January 2021 to August 2021. Ethical approval for this study (KY2020-150–02) was provided by the Institutional Review Board of Beijing Tiantan Hospital, Capital Medical University, Beijing, China on January 17, 2021, and written informed consent was obtained from all patients. The trial was registered before patient enrollment at clinicaltrials.gov (31/12/2020, NCT04691128). The report follows the guideline for reporting parallel group randomized Consolidated Standards of Reporting Trials (CONSORT) 2010.

### Participants

We enrolled patients between 18 and 60 years of age who had an American Society of Anesthesiologists (ASA) physical status of I to III and were scheduled to undergo craniotomy for supratentorial tumors with general anesthesia. Exclusion criteria were as follows: patients with preoperative brain imaging (magnetic resonance imaging, MRI) with midline shifts over 5 mm [13]; patients scheduled for electrophysiological monitoring; patients with a history of a related anesthetic allergy; patients with a Glasgow Coma Scale score < 15; patients with histories of cerebral vascular diseases or uncontrolled cardiopulmonary diseases; patients with a body mass index (BMI) > 30 kg/m^2^; patients scheduled for retaining tracheal intubation in postoperative; and patients who were unable to comprehend and cooperate with the examination.

### Randomization and blinding

We randomly assigned patients in a 1:1 ratio to the desflurane group or TIVA group. The randomization sequence was previously computer-generated and preserved in sealed opaque envelopes. The allocation was concealed until the day of surgery. Patients, the outcome assessors and the nursing team were blinded to group assignments. The attending anesthesiologists were aware of group assignments owing to the nature of the intervention.

### Anesthesia and monitoring

After entering the operating room, all patients received standard ASA monitors. Intraoperative monitoring included electrocardiography (ECG), noninvasive blood pressure, pulse oxygen saturation, end-tidal carbon dioxide (ETCO_2_), the bispectral index (BIS), nasopharyngeal temperature and urine output. An artery catheter was inserted for invasive blood pressure monitoring and blood sampling.

All patients were premedicated with 0.05 mg/kg of midazolam intravenously 15 min before anesthesia induction in the operating room. After preoxygenation, anesthesia was induced with 0.3–0.5 µg/kg of sufentanil, 1–3 mg/kg of propofol, and 0.2 mg/kg of cisatracurium. After tracheal intubation, mechanical ventilation was established with a tidal volume of 6–8 ml/kg, a fraction of inhaled oxygen of 60%, a fresh flow of 1 L/min in a semi-closed circuit, and the ventilatory frequency was adjusted between 12–15/min to maintain mild hyperventilation (PaCO_2_: 30–35 mmHg). Dexamethasone (5 mg) and ondansetron (8 mg) were administered after induction to prevent postoperative nausea and vomiting (PONV).

In the desflurane group, anesthesia was maintained with 0.8–1.0 minimum alveolar concentration (MAC) of desflurane combined with 0.05–0.2 µg·kg^−1^·min^−1^ of remifentanil. In the TIVA group, anesthesia was maintained with 6–8 mg·kg^−1^·h^−1^ of propofol combined with 0.05–0.2 µg·kg^−1^·min^−1^ of remifentanil. In both groups, sufentanil (5–10 µg) was given to alleviate potential stress responses when the headpins were placed and a scalp incision was performed. The last sufentanil bolus (0.1 µg/kg) was added when suturing dura mater [14]. Crystalloids were given as maintenance fluid, and colloids were used as per standard institutional practice and were left at the discretion of the attending anesthesiologist. Fluids management is based on routine hemodynamic monitoring: to maintain MAP ≥ 65 mmHg; to maintain HCT ≥ 30%; to ensure urinary output ≥ 0.5 ml/kg/h. Mannitol was not given prophylactically to avoid interfering with the assessment of brain relaxation. After the bone flap removal but before dura opening, if the neurosurgeon was concerned about excessive dural tension, rescue treatment including mannitol dehydration, hyperventilation, and the reverse Trendelenburg position would be given for safety concerns. Desflurane and propofol were reduced according to the BIS and hemodynamic parameters at the beginning of skin dressing and stopped once the surgery ended. In the desflurane group, the fresh gas flow was increased to minute ventilation to wash out residual anesthetic gas completely when the skull clamp was removed. In both groups, neostigmine (1–2 mg) and atropine (0.5–1 mg) were administered to antagonize residual muscle relaxation if necessary.

The dosage of anesthetics was adjusted to maintain BIS between 40 and 55. The mean arterial pressure (MAP) was maintained at a level of ± 20% compared to baseline. Baseline MAP was defined as the average value of the first three MAP measurements from midazolam administration to induction. If MAP exceeded this range, fluid infusion would be adjusted, and vasopressor or vasodilator would be given accordingly. The intraoperative nasopharyngeal temperature was maintained between 36 °C and 37 °C.

### Measurements

Preoperative peritumoral edema was evaluated by measuring the shortest straight line between the tumor edema margins on the T2 sequence of MRI (Supplementary Table 1) [15].

The primary outcome was the proportion of satisfactory brain relaxation, which was assessed by four designated neurosurgeons using a standardized 4-point scale upon dura opening. Brain relaxation was dichotomized into satisfactory (grades 1 and 2, representing perfectly and adequate relaxation) or unsatisfactory (grades 3 and 4, representing a firm and bulging brain) (Supplementary Table 2) [16].

Secondary outcomes included emergence and extubation times, recovery of cognitive function and postoperative complications. Emergence time was defined as the time from drug discontinuation to eyes opening. Extubation time was defined as the time from anesthetic discontinuation to tracheal extubation. The recovery of cognitive function was assessed with the Short Orientation Memory Concentration Test (SOMCT, scores ranging from 0 to 28, with higher scores indicating better cognitive function) by the attending anesthesiologists at 15 min and 30 min after extubation [17] (Supplementary Table 3). Postoperative complications included hypertension, tachycardia, agitation and PONV during the recovery period (operating room and post-anesthesia care unit, PACU). Hypertension was defined as MAP > 20% from baseline. Tachycardia was defined as heart rate > 100 beats/min. Agitation was assessed with Richmond Agitation Sedation Scale (scores ranging from –5 to + 4, where score 1 and above was defined as agitation). PONV was assessed using a four-point verbal rating scale (none, mild, moderate, or severe), and was further dichotomized into non-occurrence (none) and occurrence (mild, moderate, or severe) [18].

### Statistical analysis

The PASS 15 software (NCSS, LLC, USA) was used to calculate the sample size based on the primary endpoint. According to a previous study, the proportion of satisfactory brain relaxation was about 80% in the TIVA group [4]. Based on our pre-experimental results (5/9≈55%) and considering desflurane has the most pronounced effect on cerebral vasodilatation among inhalational anesthetics, which may potentially lead to unsatisfactory brain relaxation, we hypothesized that the proportion would be 55% in the desflurane group. Taking this into account, the sample size in each group should be fifty-five to achieve a power of 80% at a two-tailed significant level of 0.05, with a drop-out rate of 5%.

The statistical analysis was performed on a modified intention-to-treat basis (ITT). For the primary outcome, analysis was also performed in the per-protocol (PP) population, excluding those who received rescue treatment before dura opening. Absolute standardized difference (ASD) was determined to identify any imbalance in baseline characteristics.

Categorical variables (including the primary outcome) were presented as counts (percentages) and analyzed using χ^2^ tests. Normality was tested using the kolmogorov–Smirnov test. Continuous variables with normal distributions were presented as means with standard deviations (SD) and analyzed using student’s t-tests. Continuous variables with nonnormal distributions were presented as medians with interquartile ranges (IQR) and analyzed using Mann–Whitney U tests. Differences in the consecutive measurements of the intraoperative MAP values between the groups were evaluated using repeated-measures analysis of variance (RM-ANOVA). Furthermore, A multivariate logistic regression was used to identify predictors for satisfactory brain relaxation. Variables were initially included in univariate analysis and were entered into the multivariate model if they had a univariate *P* value of less than 0.1. The anesthesia regimen (desflurane anesthesia vs. TIVA) was forced into the multivariate model. The Hosmer–Lemeshow test was used to test the goodness of fit for the logistic regression model.

A two-sided *P* < 0.05 was considered statistically significant. Multiple comparisons were corrected to maintain the overall significance level. SPSS V.25.0 software (Chicago, IL, USA) was used for all statistical analyses.

## Results

**Fig. 1:**
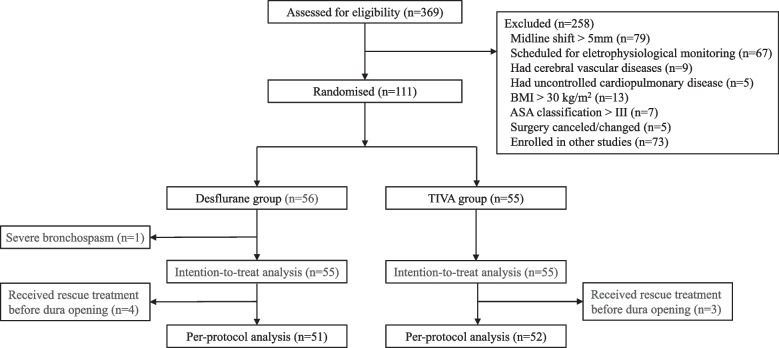
CONSORT flow diagram. Abbreviations: BMI, body mass index; TIVA, total intravenous anesthesia

From January 2021 to August 2021, 369 consecutive patients with supratentorial brain tumors scheduled for elective craniotomy were screened for eligibility. A total of 111 patients were enrolled and randomly assigned to either the desflurane group (*n* = 56) or the TIVA group (*n* = 55). One patient in the desflurane group was excluded because of severe bronchospasm after anesthesia induction. A total of 110 patients were eventually included in the modified intention-to-treat analysis (55 patients in each group) (Fig. 1). The baseline data, including demographics, preoperative comorbidities, and tumor characteristics, were similar in the two groups (Table 1).

**Table 1:** Demographics and baseline characteristics

	**Desflurane-remifentanil** **(*n* = 55)**	**Propofol-remifentanil** **(*n* = 55)**	**ASD**
Age, y	46 (35–52)	48 (36–56)	0.150
Male sex	22 (40)	21 (38)	0.041
BMI, kg/m^2^	25 (22–26)	24 (21–26)	0.276
Preoperative comorbidities			
Hypertension	11 (20)	18 (33)	0.291
Diabetes	4 (7)	6 (11)	0.118
Charlson Comorbidity Index	1 (0–2)	1 (0–2)	0
ASA physical status			
I	10 (18)	10 (18)	0
II	43 (78)	44 (80)	0.044
III	2 (4)	1 (2)	0.111
SOMCT score	26 (24–28)	26 (24–28)	0
Tumor size, cm^3^	8 (4–14)	11 (5–21)	0.147
Tumor classification			
Glioma	19 (35)	23 (42)	0.108
Meningioma	28 (51)	25 (46)	0.108
Metastatic	1 (2)	1 (2)	0
Other	7 (13)	6 (11)	0.056
Tumor site			
Frontal	22 (40)	23 (42)	0.037
Parietal	8 (15)	7 (13)	0.053
Temporal	11 (20)	14 (26)	0.131
Occipital	4 (7)	5 (9)	0.066
Others	10 (18)	6 (10)	0.208
Preoperative edema			
0: No signs	34 (62)	34 (62)	0
I: Mild	13 (24)	17 (31)	0.165
II: Moderate	6 (11)	3 (5)	0.198
III: Severe	2 (3)	1 (2)	0.111

Data are reported as no. (%) or median (IQR). 1.96*√((n1 + n2)/n1n2) = 0.374, and all the ASD in the table is smaller than 0.374

*Abbreviations: ASD* Absolute standardized difference, *ASA* American society of anesthesiologists, *BMI* Body mass index, *IQR* Interquartile range, *SOMCT* Short orientation memory concentration test

Intraoperative factors that may affect brain relaxation were well balanced between the two groups except for MAP (Table 2). Patients assigned to the desflurane group showed a more significant decrease in MAP than those assigned to the TIVA group after induction (Supplementary Fig. 1; *P* = 0.012 by RM-ANOVA). Accordingly, the desflurane group required more vasopressor medications while the patients in the TIVA group required more vasodilator medications. The requirements for rescue treatments after bone flap removal were similar between the two groups [4 (7%) vs. 3 (5%), *P* = 1.000]. Total remifentanil consumption was significantly lower in the desflurane group (mean difference: -1.3 mg, *P* < 0.0001).

**Table 2:** Intraoperative parameters

	**Desflurane-remifentanil** **(*n* = 55)**	**Propofol-remifentanil** **(*n* = 55)**	***P***
Head position^a^			0.265
Neutral	23 (42)	21 (38)	
≤ 45°	26 (47)	22 (40)	
> 45°	6 (11)	12 (22)	
At dura opening			
MAP, mmHg	76 ± 7	84 ± 15	0.002
Fluid input, ml	1010 ± 220	1070 ± 230	0.156
Fluid output, ml	450 (250–550)	500 (250–650)	0.084
PaCO_2_, mmHg	34 (33–35)	33 (32–35)	0.276
Rescue treatment^b^	4 (7)	3 (5)	1.000
Intraoperative medications			
Propofol, mg	/	1300 (1100–1800)	
Desflurane, MAC-hour	0.8 ± 0.03	/	
Sufentanil, μg	45 (40–50)	45 (40–50)	0.712
Remifentanil, mg	1.1 ± 0.5	2.4 ± 0.9	< 0.0001
Cisatracurium, mg	18.4 ± 5.3	17.6 ± 5.0	0.324
Use of vasopressors	11 (20)	4 (7)	0.052
Use of vasodilators	0	17 (31)	< 0.0001
Surgery duration, min	194 (145–250)	190 (155–230)	0.895
Anesthesia duration, min	230 (180–280)	228 (185–265)	0.967
Temperature, ℃	36.1 ± 0.1	36.2 ± 0.2	0.080

Data are reported as no. (%), mean ± SD or median (IQR). *P* < 0.05 was considered statistically significant

*Abbreviations*: *IQR* Interquartile range, *MAP* Mean arterial pressure, *MAC* Minimum alveolar concentration, *PaCO*_*2*_ Arterial partial pressure of carbon dioxide, *SD* Standard deviation

^a^Taking the standard anatomical posture as a reference, the patients were divided into three groups according to whether the head was deflected and whether the degree of deflection exceeded 45°

^b^Rescue treatment included hyperventilation, mannitol rescue and reverse Trendelenburg position

### Primary outcome

**Fig. 2:**
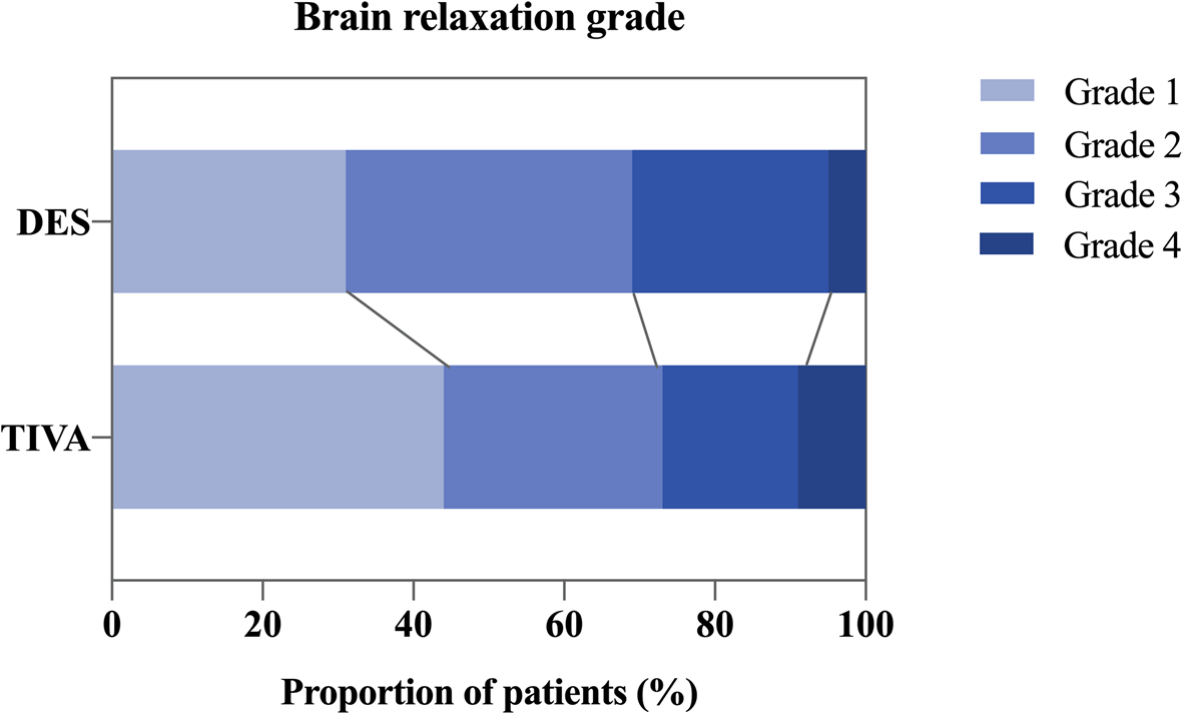
Brain relaxation grade. Abbreviations: DES, desflurane anesthesia; TIVA, total intravenous anesthesia

The proportion of satisfactory brain relaxation was similar between the two groups: 69% in the desflurane group and 73% in the TIVA group (RR: 0.950, 95% CI, 0.748–1.207; *P* = 0.675) (Table 3; Fig. 2). The per-protocol analysis yielded a similar result (RR: 0.939, 95% CI, 0.733–1.204; *P* = 0.619). Univariate and multivariate analysis did not show a significant effect of the anesthesia regimen on brain relaxation (Table 4 and Supplementary Table 4). Multivariate analysis showed that peritumoral edema (OR: 0.328 per increased edema grade, 95% CI, 0.164–0.654; *P* = 0.002) and occipital tumors (OR: 0.067, 95% CI, 0.011–0.416; *P* = 0.004) were independent predictors for unsatisfactory brain relaxation (Table 4).

**Table 3:** The Primary outcome and secondary outcomes

	**Desflurane-remifentanil** **(*n* = 55)**	**Propofol-remifentanil** **(*n* = 55)**	***P***
**Primary outcome**			
Satisfactory brain relaxation			
ITT analysis	38 (69)	40 (73)	0.675^a^
PP analysis	35 (69)	38 (73)	0.619^a^
**Secondary outcome**			
Emergence time, min	10 (8–13)	13 (10–20)	< 0.001^b^
Extubation time, min	13 (10–18)	17 (13–23)	< 0.001^b^
SOMCT score			
15 min after extubation	16 (0–24)	0 (0–20)	0.003^b^
30 min after extubation	20 (4–26)	7 (0–23)	0.063^b^
Recovery complications			
Hypertension	9 (16)	4 (7)	0.140^a^
Tachycardia	22 (40)	9 (16)	0.006^a^
Agitation	5 (9)	10 (18)	0.165^a^
PONV	16 (29)	6 (11)	0.017^a^

Data are reported as no. (%) or median (IQR). *P* < 0.05 was considered statistically significant. 103 patients were included in the PP analysis (51 in the desflurane group and 52 in the TIVA group)

*Abbreviations*: *ITT* Intention-to-treat, *PP* Per-protocol, *PONV* Postoperative nausea and vomiting, *SOMCT* Short orientation memory concentration test

^a^χ^2^ test

^b^Mann–Whitney U tests

**Table 4:** Multivariate logistic regression analysis of satisfactory brain relaxation

Parameters	Multivariable OR	95% CI	*P*
Desflurane (vs. TIVA)	0.719	0.256–2.019	0.531
Location			
Frontal	Ref^a^	Ref^a^	Ref^a^
Parietal	6.158	0.534–71.067	0.145
Temporal	1.174	0.356–3.871	0.792
Occipital	0.067	0.011–0.416	0.004
Others	3.467	0.380–31.614	0.270
Peritumoral edema	0.328	0.164–0.654	0.002
Tumor size, cm^3^	0.976	0.946–1.008	0.143

*P* = 0.699 > 0.05 in the Hosmer–Lemeshow test indicates a good fit for the model

*Abbreviations*: *CI* Confidence interval, *OR* Odds ratio, *TIVA* Total intravenous anesthesia

^a^Ref indicates the reference parameter in the subgroup analysis

### Secondary outcomes

The emergence time and extubation time in the desflurane group were shorter than those in the TIVA group (10 [8–13] min vs. 13 [10–20] min, *P* < 0.001), (13 [10–18] min vs. 17 [13–23] min, *P* < 0.001). In addition, patients assigned to the desflurane group had higher median scores of SOMCT at 15 min after extubation (16 [0–24] vs. 0 [0–20], *P* = 0.003) During the recovery period, patients assigned to the desflurane group experienced more tachycardia (22 [40%] vs. 9 [16%], *P* = 0.006) and PONV (16 [29%] vs. 6 [11%], *P* = 0.017) The incidence of hypertension and agitation did not differ between the two groups.

## Discussion

Our study suggested that desflurane anesthesia and TIVA provide similar brain relaxation in patients undergoing craniotomy for supratentorial tumors without intracranial hypertension.

Several studies have demonstrated propofol decrease ICP by reducing CBF and CBV simultaneously [19, 20], while desflurane anesthesia dose-dependently increases ICP by promoting cerebral vasodilation [9]. However, no difference in brain relaxation among different anesthesia regimens has been demonstrated in several clinical trials [10–12]. It should be noted that these studies were not designed to investigate the effect of anesthesia regimens on brain relaxation and the routine use of mannitol may largely influence the evaluation of brain relaxation. Our study fully considered the factors that may influence brain relaxation, including tumor characteristics, mannitol, fluid balance and PaCO_2_, and provides more sufficient evidence that the theoretical cerebral vasodilation effect of desflurane does not lead to unsatisfactory brain relaxation compared with TIVA in clinical practice.

Multiple factors may account for our major finding. First, the cerebral vasodilation effect of desflurane is dose-dependent. Low-dose desflurane decreases global CBF by suppressing cerebral metabolism. As the concentration increases, the direct vasodilation effect begins to dominate and may increase CBF, while these effects were mainly observed at concentrations of 1.0 MAC and above [8]. In our study, a MAC of 0.8 of desflurane combined with opioid agents achieved a sufficient depth of anesthesia, therefore, the potential effect of cerebral vasodilation may not appear. Second, the impact of different anesthesia regimens on brain relaxation may be associated with the patient's intracranial status. Preethi et al.[21]. reported that TIVA is superior to inhalational anesthesia (isoflurane) in providing brain relaxation in patients with severe traumatic brain injury. Severe traumatic brain injury is often accompanied by the impairment of cerebrovascular autoregulation and exhausted intracranial compliance. Therefore, the choice of anesthesia regimen may play a pivotal role in controlling ICP and promoting brain relaxation. However, our study was conducted in patients with midline shifts less than 5 mm who have maintained intracranial compliance, such patients may preserve constant ICP while receiving inhalational anesthetic [13]. Third, it is well known that carbon dioxide (CO_2_) is a powerful modulator of cerebral vasomotor tone, and hypocapnia leads to cerebral vasoconstriction [22]. In our study, mild hyperventilation (PaCO_2_ of 30 to 35 mmHg) was maintained during surgery following the clinical management routine. Consequently, cerebral vasoconstriction secondary to hypocapnia may mask the direct vasodilatation effect of desflurane. Moreover, in our study, the cerebral hemodynamic effect of desflurane was further complicated by the significant decrease in MAP. It cannot be ignored that similar brain relaxation may occur as a consequence of a decrease in cerebral perfusion pressure. Lastly, fluid balance is a crucial factor affecting brain relaxation, and fluid overload can exacerbate cerebral edema. Our study used uniform fluid management criteria, and the results showed that the fluid input and output were comparable between the two groups at dural opening. Therefore, the interference of fluid balance on brain relaxation assessment was well controlled in our study.

The multivariate analysis revealed that peritumoral edema is associated with unsatisfactory brain relaxation, which is consistent with previous findings [16, 23]. Moreover, our study also found that tumor location (occipital tumors) is a risk factor for unsatisfactory brain relaxation. Occipital tumor surgery often requires twisting of the patient's neck to facilitate the operation, lateral flexion or torsion may obstruct venous drainage, with resultant unsatisfactory brain relaxation. Therefore, effective intervention should be carried out positively in these patients to achieve better brain relaxation.

The present study also found that, compared with TIVA, desflurane anesthesia provides patients with faster emergence and better recovery of cognitive function. In clinical practice, rapid recovery is desirable in neurosurgery because it allows for early neurological assessment and prompt detection of potential complications, such as hematoma formation, acute cerebral infarction, and neurological deficits. This contributes to rapid diagnosis and intervention and may improve patients’ clinical outcomes [24].

Recovery complications were similar between the two groups, except for PONV and tachycardia. Patients assigned to the desflurane group experienced more PONV than those in the TIVA group even though we administered 5 mg of dexamethasone combined with 8 mg of ondansetron to prevent PONV. A recent review suggested that 8 mg of dexamethasone may significantly enhance the antiemetic effect, which could be tested in future studies [25]. In addition, more tachycardia episodes were observed in the desflurane group than in the TIVA group during the recovery period, we speculate that it may be associated with fewer opioids administration, and earlier perception of adverse stimulations after rapid emergence, or uncomfortable feelings caused by PONV.

Our study has several limitations. First, we did not supplement any objective measures to evaluate brain relaxation, such as subdural pressure and cerebrospinal fluid pressure monitoring, but only a subjective evaluation by the neurosurgeons. However, the standardized 4-point scale is the most practical and accessible measurement to evaluate brain relaxation, and it has been widely applied in many clinical studies [10, 16, 26–29]. Second, we only enrolled patients without severe intracranial hypertension, so the results cannot be extrapolated to patients with low cerebral compliance. Third, our study may have potential bias. The attending anesthesiologists were aware of the group allocation, but the neurosurgeons who assessed brain relaxation were blinded.

## Conclusions

In conclusion, among patients undergoing elective craniotomy without severe intracranial hypertension, desflurane anesthesia and TIVA provide similar brain relaxation assessed by the neurosurgeons using a 4-point scale. Desflurane anesthesia provides faster recovery but is associated with increased PONV and tachycardia during the recovery period. Therefore, we should fully balance the strengths and weaknesses of desflurane in clinical practice and optimize the management strategy to benefit patients undergoing neurosurgery.

## Supplementary Information

**Additional file 1:** **Figure 1.** Jpg Intraoperative Mean Arterial Pressure. Differences in the intraoperative MAP values between the groups were evaluated using RM-ANOVA (*P* = 0.012). T0, before anesthesia induction; T1, 1 hour after anesthesia induction; T2, dura opening; T3, 2 hours after anesthesia induction; T4, at the end of surgery; T5, emergence. Abbreviations: MAP, mean arterial pressure; RM-ANOVA, repeated-measures analysis of variance

**Additional file 2:** **Table 1.** Steinhoff classification.

**Additional file 3:** **Table 2.** Brain relaxation 4-point scale.

**Additional file 4:** **Table 3.** Short Orientation Memory Concentration Test.

**Additional file 5:** **Table 4.** Univariate Logistic Regression Analysis of Satisfactory Brain Relaxation.

## Data Availability

The datasets used and analyzed during the current study are available from the corresponding author (Ruquan Han: ruquan.han@ccmu.edu.cn) upon reasonable request.

## Abbreviations

ASA: American Society of Anesthesiologists
ASD: Absolute standardized difference
BMI: Body mass index
BIS: Bispectral index
CBF: Cerebral blood flow
CBV: Cerebral blood volume
CI: Confidence interval
CONSORT: Consolidated Standards of Reporting Trials
ECG: Electrocardiography
ETCO_2_: End-tidal carbon dioxide
ICP: Intracranial pressure
IQR: Interquartile range
ITT: Intention-to-treat
MAC: Minimum alveolar concentration
MAP: Mean arterial pressure
MRI: Magnetic resonance imaging
OR: Odds ratio
PaCO_2_: Arterial pressure of carbon dioxide
PONV: Postoperative nausea and vomiting
PACU: Post-anesthesia care unit
PP: Per-protocol
RM-ANOVA: Repeated-measures analysis of variance
SOMCT: Short Orientation Memory Concentration Test
SD: Standard deviations
TIVA: Total intravenous anesthesia

## References

[1] Li J, Gelb AW, Flexman AM, Ji F, Meng L (2016). Definition, evaluation, and management of brain relaxation during craniotomy. Br J Anaesth.

[2] Hans P, Bonhomme V (2006). Why we still use intravenous drugs as the basic regimen for neurosurgical anesthesia. Curr Opin Anaesthesiol.

[3] Petersen KD, Landsfeldt U, Cold GE, Petersen CB, Mau S, Hauerberg J (2003). Intracranial pressure and cerebral hemodynamic in patients with cerebral tumors: a randomized prospective study of patients subjected to craniotomy in propofol-fentanyl, isoflurane-fentanyl, or sevoflurane-fentanyl anesthesia. Anesthesiology.

[4] Citerio G, Pesenti A, Latini R, Masson S, Barlera S, Gaspari F (2012). A multicentre, randomised, open-label, controlled trial evaluating equivalence of inhalational and intravenous anesthesia during elective craniotomy. Eur J Anaesthesiol.

[5] Santra S, Das B (2009). Subdural Pressure and Brain Condition During Propofol Vs Isoflurane - Nitrous Oxide Anesthesia in Patients Undergoing Elective Supratentorial Tumor Surgery. Indian J Anaesth.

[6] Chui J, Mariappan R, Mehta J, Manninen P, Venkatraghavan L (2014). Comparison of propofol and volatile agents for maintenance of anesthesia during elective craniotomy procedures: systematic review and meta-analysis. Can J Anaesth.

[7] Magni G, Rosa IL, Melillo G, Savio A, Rosa G (2009). A Comparison Between Sevoflurane and Desflurane Anesthesia in Patients Undergoing Craniotomy for Supratentorial Intracranial Surgery. Anesth Analg.

[8] Bedforth NM, Girling KJ, Skinner HJ, Mahajan RP (2001). Effects of desflurane on cerebral autoregulation. Br J Anaesth.

[9] Holmstrom A, Åkeson J (2004). Desflurane Increases Intracranial Pressure More and Sevoflurane Less Than Isoflurane in Pigs Subjected to Intracranial Hypertension. J Neurosurg Anesthesiol.

[10] Bhagat H, Sharma T, Mahajan S, Kumar M, Saharan P, Bhardwaj A (2021). Intravenous versus inhalational anesthesia trial for outcome following intracranial aneurysm surgery: A prospective randomized controlled study. Surg Neurol Int.

[11] Bhardwaj A, Bhagat H, Grover VK, Panda NB, Jangra K, Sahu S (2018). Comparison of propofol and desflurane for postanaesthetic morbidity in patients undergoing surgery for aneurysmal SAH: a randomized clinical trial. J Anesth.

[12] Sharma N, Wig J, Mahajan S, Chauhan R, Mohanty M, Bhagat H (2020). Comparison of postoperative cognitive dysfunction with the use of propofol versus desflurane in patients undergoing surgery for clipping of aneurysm after subarachnoid hemorrhage. Surg Neurol Int.

[13] Fraga M, Rama-Maceiras P, Rodiño S, Aymerich H, Pose P, Belda J (2003). The Effects of Isoflurane and Desflurane on Intracranial Pressure, Cerebral Perfusion Pressure, and Cerebral Arteriovenous Oxygen Content Difference in Normocapnic Patients with Supratentorial Brain Tumors. Anesthesiology.

[14] Xing Y, Lin N, Han R, Bebawy JF, Peng Y, Li J (2020). Sevoflurane versus PRopofol combined with Remifentanil anesthesia Impact on postoperative Neurologic function in supratentorial Gliomas (SPRING): protocol for a randomized controlled trial. BMC Anesthesiol.

[15] Kazner E, Lanksch W, Steinhoff H, Wilske J (1975). Computerized axial tomography of the skull - diagnostic possibilities and clinical results (author's transl). Fortschr Neurol Psychiatr Grenzgeb.

[16] Li S, Sun H, Liu X, Ren X, Hao S, Zeng M (2020). Mannitol Improves Intraoperative Brain Relaxation in Patients With a Midline Shift Undergoing Supratentorial Tumor Surgery: A Randomized Controlled Trial. J Neurosurg Anesthesiol.

[17] Bilotta F, Doronzio A, Cuzzone V, Caramia R, Rosa G (2009). PINOCCHIO Study Group. Early Postoperative Cognitive Recovery and Gas Exchange Patterns After Balanced Anesthesia With Sevoflurane or Desflurane in Overweight and Obese Patients Undergoing Craniotomy A Prospective Randomized Trial. J Neurosurg Anesthesiol.

[18] Ziemann-Gimmel P, Goldfarb AA, Koppman J, Marema RT (2014). Opioid-free total intravenous anesthesia reduces postoperative nausea and vomiting in bariatric surgery beyond triple prophylaxis. Br J Anaesth.

[19] Vandesteene A, Trempont V, Engelman E, Deloof T, Focroul M, Schoutens A (1988). Effect of propofol on cerebral blood flow and metabolism in man. Anesthesia.

[20] Lagerkranser M, Stånge K, Sollevi A (1997). Effects of propofol on cerebral blood flow, metabolism, and cerebral autoregulation in the anesthetized pig. J Neurosurg Anesthesiol.

[21] Preethi J, Bidkar PU, Cherian  A, Dey A, Srinivasan S, Adinarayanan S (2021). Comparison of total intravenous anesthesia vs. inhalational anesthesia on brain relaxation, intracranial pressure, and hemodynamics in patients with acute subdural hematoma undergoing emergency craniotomy: a randomized control trial. Eur J Trauma Emerg Surg.

[22] Zhang Z, Guo Q, Wang E (2019). Hyperventilation in neurological patients: from physiology to outcome evidence. Curr Opin Anaesthesiol.

[23] Bedford RF, Morris L, Jane JA (1982). Intracranial hypertension during surgery for supratentorial tumor: correlation with preoperative computed tomography scans. Anesth Analg.

[24] Prabhakar H, Singh GP, Mahajan C, Kapoor I, Kalaivani  M, Anand V (2016). Anand V. Intravenous versus inhalational techniques for rapid emergence from anesthesia in patients undergoing brain tumor surgery. Cochrane Database Syst Rev.

[25] Myles PS, Corcoran T (2021). Benefits and Risks of Dexamethasone in Noncardiac Surgery. Anesthesiology.

[26] Lima MF, Neville IS, Cavalheiro S, Bourguignon DC, Pelosi P, Malbouisson LMS (2019). Balanced Crystalloids Versus Saline for Perioperative Intravenous Fluid Administration in Children Undergoing Neurosurgery: A Randomized Clinical Trial. J Neurosurg Anesthesiol.

[27] Rozet I, Tontisirin N, Muangman S, Vavilala MS, Souter MJ, Lee LA (2007). Effect of Equiosmolar Solutions of Mannitol versus Hypertonic Saline on Intraoperative Brain Relaxation and Electrolyte Balance. Anesthesiology.

[28] Seo H, Kim E, Jung H, Lim YJ, Kim JW, Park CK (2017). A prospective randomized trial of the optimal dose of mannitol for intraoperative brain relaxation in patients undergoing craniotomy for supratentorial brain tumor resection. J Neurosurg.

[29] Quentin C, Charbonneau S, Moumdjian R, Lallo A, Bouthilier A, Fournier-Gosselin MP (2013). A comparison of two doses of mannitol on brain relaxation during supratentorial brain tumor craniotomy: a randomized trial. Anesth Analg.

