# Supplementary figures and images for: Brain relaxation using desflurane anesthesia and total intravenous anesthesia in patients undergoing craniotomy for supratentorial tumors: a randomized controlled study
Source: BMC Anesthesiol. 2023 Jan 10;23:15. doi: 10.1186/s12871-023-01970-z (PMC9830805; doi:10.1186/s12871-023-01970-z)

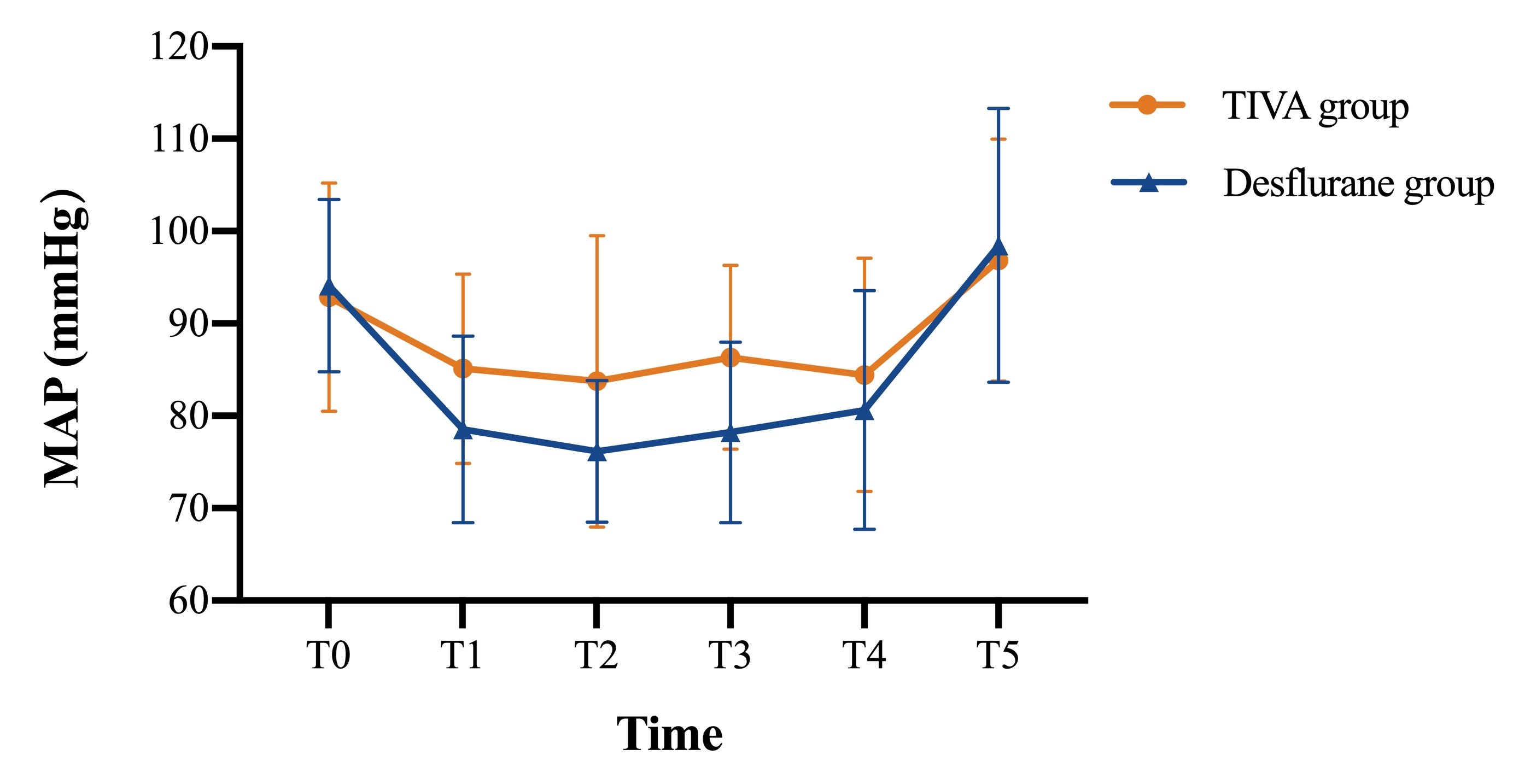

Supplement: Supplementary file 1 — Additional file 1: Figure 1. Jpg Intraoperative Mean Arterial Pressure. Differences in the intraoperative MAP values between the groups were evaluated using RM-ANOVA (P = 0.012). T0, before anesthesia induction; T1, 1 hour after anesthesia induction; T2, dura opening; T3, 2 hours after anesthesia induction; T4, at the end of surgery; T5, emergence. Abbreviations: MAP, mean arterial pressure; RM-ANOVA, repeated-measures analysis of variance [file 12871_2023_1970_MOESM1_ESM.jpg]
