# Supplementary material for: Brain relaxation using desflurane anesthesia and total intravenous anesthesia in patients undergoing craniotomy for supratentorial tumors: a randomized controlled study
Source: BMC Anesthesiol. 2023 Jan 10;23:15. doi: 10.1186/s12871-023-01970-z (PMC9830805; doi:10.1186/s12871-023-01970-z)
Supplement: Supplementary file 2 — Additional file 2: Table 1. Steinhoff classification. [file 12871_2023_1970_MOESM2_ESM.docx]

**Supplementary Table 1. Steinhoff classification**

| **Grade** | **Edema** | **Severity** |
| --- | --- | --- |
| 0 | No signs of edema | No sign |
| I | Edema limited to 2 cm | Mild |
| II | Edema more than 2 cm and limited to the ipsilateral hemisphere | Moderate |
| III | Edema extending to the contralateral hemisphere | Severe |
