# Supplementary material for: Brain relaxation using desflurane anesthesia and total intravenous anesthesia in patients undergoing craniotomy for supratentorial tumors: a randomized controlled study
Source: BMC Anesthesiol. 2023 Jan 10;23:15. doi: 10.1186/s12871-023-01970-z (PMC9830805; doi:10.1186/s12871-023-01970-z)
Supplement: Supplementary file 3 — Additional file 3: Table 2. Brain relaxation 4-point scale. [file 12871_2023_1970_MOESM3_ESM.docx]

**Supplementary Table 2. Brain relaxation 4-point scale**

| **Grade** | **Description** | **Satisfactory/Unsatisfactory** |
| --- | --- | --- |
| 1 | Perfectly relaxed; just as normal brain tension | Satisfactory brain relaxation |
| 2 | Adequate relaxed; mild brain swelling, acceptable |  |
| 3 | Firm brain; no therapy required | Unsatisfactory brain relaxation |
| 4 | Bulging brain; requiring treatment. |  |

The evaluations of brain relaxation were conducted by four designated neurosurgeons who expert in the intracranial tumor. The grading method was fully explained to the evaluators before assessment.
