# Supplementary material for: Brain relaxation using desflurane anesthesia and total intravenous anesthesia in patients undergoing craniotomy for supratentorial tumors: a randomized controlled study
Source: BMC Anesthesiol. 2023 Jan 10;23:15. doi: 10.1186/s12871-023-01970-z (PMC9830805; doi:10.1186/s12871-023-01970-z)
Supplement: Supplementary file 4 — Additional file 4: Table 3. Short Orientation Memory Concentration Test. [file 12871_2023_1970_MOESM4_ESM.docx]

**Supplementary Table 3. Short Orientation Memory Concentration Test**

| **Questions** | **Scores** |
| --- | --- |
| What is the current year? | Correct answer scores 4, incorrect answer scores 0 |
| What is the current month? | Correct answer scores 3, incorrect answer scores 0 |
| What time is it? | Correct answer scores 3, incorrect answer scores 0 |
| Count backwards from 20 to 1^a^ | Max score 4 |
| Say the months of the year backwards ^a^ | Max score 4 |
| Repeat the information given in the preceding sentence ^b^ | Max score 10 |

At the beginning of each test (preoperative visit; 15, 30 min after extubation) the attending anesthesiologist tells the patient the current time of day, day, and year; gives 5 items of information (name, surname, street number and name, and town); and 1 minute later begin to test.

These 6 variables yield scores ranging from 0 to 28, with higher scores indicating better cognitive function and scores more than 20 were considered normal.

^a^ One point less for each mistake; more than 4 mistakes still score 0.

^b^ Each mistake takes 2 points away (name, surname, street name, street number, and town).
