# Supplementary material for: Brain relaxation using desflurane anesthesia and total intravenous anesthesia in patients undergoing craniotomy for supratentorial tumors: a randomized controlled study
Source: BMC Anesthesiol. 2023 Jan 10;23:15. doi: 10.1186/s12871-023-01970-z (PMC9830805; doi:10.1186/s12871-023-01970-z)
Supplement: Supplementary file 5 — Additional file 5: Table 4. Univariate Logistic Regression Analysis of Satisfactory Brain Relaxation. [file 12871_2023_1970_MOESM5_ESM.docx]

**Supplementary Table 4. Univariate Logistic Regression Analysis of Satisfactory Brain Relaxation.**

| **Parameters** | **Univariable OR** | **95% CI** | **P** |
| --- | --- | --- | --- |
| Desflurane (vs. TIVA) | 0.838 | 0.368-1.911 | 0.675 |
| Tumor size, cm^3^ | 0.959 | 0.930-0.990 | 0.009 |
| Location |  |  |  |
| Frontal | Ref^a^ | Ref^a^ | Ref^a^ |
| Parietal | 7.724 | 0.928-64.260 | 0.059 |
| Temporal | 1.419 | 0.489-4.116 | 0.520 |
| Occipital | 0.158 | 0.029-0.851 | 0.032 |
| Others | 8.276 | 0.999-68.553 | 0.050 |
| Type |  |  |  |
| Glioma | Ref^a^ | Ref^a^ | Ref^a^ |
| Meningioma | 0.881 | 0.363-2.136 | 0.778 |
| Metastatic | 0.395 | 0.023-6.726 | 0.521 |
| Others | 1.316 | 0.317-5.454 | 0.705 |
| Peritumoral edema | 0.337 | 0.186-0.611 | < 0.0001 |
| Fluid balance, ml | 1.001 | 1.000-1.003 | 0.154 |
| PaCO_2_, mmHg | 0.993 | 0.831-1.186 | 0.937 |

Abbreviations: CI, confidence interval; OR, odds ratio; PaCO_2_, partial pressure of carbon dioxide in artery; TIVA, total intravenous anesthesia.

^a^Ref indicates the reference parameter in the subgroup analysis.
